# Antiangiogenic therapies in urogenital malignancies: Fiction or fact?

**DOI:** 10.1007/s12254-017-0375-8

**Published:** 2017-12-04

**Authors:** Friederike Haidl, David Pfister, Axel Heidenreich, Isabel Heidegger

**Affiliations:** 1 Department of Urology, Uro-Oncology, Robot Assisted and Reconstructive Urologic Surgery, University Hospital Cologne, Kerpener Straße 62, 50937 Cologne, Germany; 2 Department of Urology, Medical University Innsbruck, Anichstraße 35, 6020 Innsbruck, Austria

**Keywords:** Prostate cancer, Testicular cancer, Penile cancer, Urothelial cancer, Antiangiogenic therapeutics

## Abstract

The use of antiangiogenic agents in cancer therapy has become an attractive target in oncological research. However, concerning the uro-oncological field, current guidelines only recommend the use of antiangiogenic agents in metastatic renal cell cancer. Yet in recent years, several approaches for sequential treatment with angiogenesis inhibitors in other urogenital malignancies apart from renal cell cancer are ongoing. Thus, the present review article aims to provide an overview about clinical studies with antiangiogenic agents in prostate-, bladder-, testicular-, as well as penile cancer patients. For this, a literature search was conducted using Medline; moreover we performed a systematic review of data presented at this year’s important urooncological meetings. Preliminary data revealed that there are several promising studies ongoing in prostate-, bladder-, testicular-, as well as penile cancer; however, larger studies should be conducted to optimize the use of antiangiogenic agents in clinical practice.

## Introduction

Tumor angiogenesis plays an important role in cancer growth and metastatic dissemination. Diffusion suffices to supply nutrients and oxygen in tumors up to 3 mm in diameter [1], while survival and growth of tumors beyond this dimension depend on the new formation of a sufficient vessel network, primarily by angiogenesis [2]. In general, angiogenesis is mainly regulated by the interaction of pro- and antiangiogenic factors such as vascular endothelial growth factor (VEGF). The inhibition of these factors is undoubtedly an attractive target for anticancer therapy successfully used as standard treatment options in several cancer entities including lung or colorectal cancer.

Concerning the uro-oncological field, antiangiogenic therapeutic strategies are clinically established only in metastatic renal cell cancer (mRCC). In general, most of the antiangiogenic agents used in mRCC are inhibitors of the VEGF pathway and their use has become an integral part of therapy for patients with mRCC also recommended as first line-treatment in the Europan Urology Association (EAU) guidelines [3].

However, besides RCC, preclinical and early clinical studies have also demonstrated that angiogenesis exerts an important therapeutic role in other urological malignancies including prostate-, bladder-, testicular-, as well as penile cancer [6, 8, 10, 14].

This review focuses on recent research findings about the role of antiangiogenic agents in the treatment of genitourinary cancers except mRCC with a special focus on recent findings presented at this year’s urooncological (GU ASCO, ASCO, ESMO and EAU) meetings.

## Penile cancer

Since the use of antiangiogenic agents has been considered to treat patients with mRCC, it is assumed that antiangiogenic therapy could also be effective in patients with penile cancer as it is also a highly vascularized tumor entity. However, as penile cancer is a rare disease with an incidence of <1/100,000 males in Europe and the USA, to our best knowledge currently no trial is investigating the impact of antiangiogenic agents in penile cancer [4].

However, based on the observation that the EGF receptor (EGFR) is almost invariably expressed in penile cancer [5] Necchi et al. investigated the efficacy of dacomitinib, a tyrosine kinase inhibitor (TKI) of human EGFR in patients with advanced or metastatic penile squamous cell carcinoma in a single arm phase 2 study (NCT01728233) [6]. In this study 26 patients with squamous cell histology, and clinical stage N2–3 or M1 disease received daily 45 mg dacomitinib. Preliminary data presented at the GU ASCO meeting revealed that 1/26 patients achieved complete remission while 7/26 had a partial remission (overall response rate [ORR] = 30,4%, 95% credibility interval 14.9–48.6%) under daily oral application of 45 mg dacomitinib. The 12-month progression-free survival (PFS) was 24.1% (95% CI: 11.1–52.3) and the 12 month overall survival (OS) was 50.7%. Mutations were found in 47% of non-responders compared to 25% of responders, among them TERT mutations (60%) were found in responders only while HRAS and BRAF mutations were found in non-responders (20%). Final results of the study are not published yet, but are expected this year [6].

## Prostate cancer

It is already known that the expression of high VEGF levels in prostate cancer cells is associated with poor prognosis [7]. Moreover, it has been shown that VEGF levels in plasma and urine of patients with metastatic castration resistant prostate cancer (mCRPC) are independent predictors of OS [7–9].

Bevacizumab is a recombinant, humanized monoclonal antibody that selectively binds VEGF‑A and prevents interaction with its receptor. A recent phase 2 trial employed bevacizumab in combination with short-term androgen deprivation therapy (ADT) in patients with hormone-sensitive recurrent prostate cancer. A total of 102 patients with hormone-sensitive recurrent prostate cancer who received ADT + bevacizumab or ADT alone were reviewed for efficacy and toxicity. Compared to the ADT-alone arm, patients treated with ADT + bevacizumab had a significant improvement in relapse-free survival (RFS) (13.3 months for ADT + bevacizumab vs 10.2 months ADT alone, *p* = 0.002). The most common grade 3 events observed in this study were hypertension which was more frequent in the ADT + bevacizumab arm (36%) [10].

Moreover, Kelly et al. conducted a phase 3 trial to investigate a potential clinical benefit in addition of bevacizumab to standard docetaxel and prednisone therapy in patients with mCRPC. A total of 1050 patients with mCRPC were enrolled among them 524 patients were assigned to the docetaxel + prednisone with bevacizumab arm while 526 patients received docetaxel + predisolone with placebo. Even though an improvement in PFS for patients treated in the docetaxel + prednisone/bevaciczumab arm (9.9 vs. 7.5 months, *P* < 0.001) could be demonstrated, combined treatment was associated with more common grade 3 or greater treatment-related toxicity compared to the control group (75.4% vs. 56.2%; *P* < 0.001). Furthermore the incidence of treatment-related deaths in the docetaxel + prednisone/bevaciczumab arm was greater (4.0% vs. 1.2%; *P* = 0.005). In addition, this trial also failed to show an improvement of OS for patients treated additionally with bevaciumab compared to docetaxel + prednisolone monotherapy (22.6 vs. 21.5 months, *P* = 0.181; [11]).

An additional phase 3 study investigated the impact of aflibercept, an inhibitor of the VEGF receptor (VEGF trap). A total of 1224 patients with mCRPC were randomized: 612 were assigned to the docetaxel + prednisone + aflibercept arm, while 612 only received docetaxel + prednisone with a placebo. As already described in the preceding study with bevacizumab the combination of aflibercept with docetaxel and prednisone did not increase OS (22.1 months in the aflibercept group vs. 21.2 months in the placebo group) but increased toxicity [12].

To summarize, these findings suggest that adding antiangiogenic agents to docetaxel and prednisone in men with mCRPC has no clinical benefit.

## Testicular cancer

In general, therapeutic responses to chemotherapy in germ cells tumors are excellent. However, in case of tumor relapse or primary chemoresistant germ cell tumors, the cure rates and therapeutic options are limited. Regarding the knowledge of increased VEGF expression in germ cell tumors [13] recent trials focus on antiangiogenic factors and their potential activity in germ cell tumors as new therapeutic option [14–17].

In this context pazopanib, a multitarget TKI against VEGFR 1–3, platelat derived growth factor (PDGFR) and c-KIT was used in a single-arm phase 2 study for patients with refractory germ cell tumors after at least failure of ≥2 platinum-based regimens. A total of 43 patients were enrolled in this study where pazopanib was administered daily. The primary aim was to evaluate the proportion of patients who are progression-free after 3 months of pazopanib. Secondary measurements were the safety and tolerability of pazopanib, response rate as well as assessment of OS rates. Data revealed that the 3‑month PFS probability was 12.8%, and the median PFS duration was 2.5 months. Interestingly, seminomatous histology was associated with shorter PFS. After 4 weeks of treatment, 70.3% of the patients showed decreased serum tumor markers. Two patients (4.7%) had a confirmed partial remission, in 19 (44.2%) cases stable disease was reached and 16 patients (37.2%) progressed. The 12-month OS probability was 28.5% and the median OS duration was 5.3 months. The number of prior regimens, the presence of liver, bone, or brain metastasis and alpha feto protein (AFP) elevation were associated with a poorer OS. Grade 3 adverse events were seen in 6 patients (13%). In addition it was possible to identify new missense mutations in tissue of 3 patients, who had a good response to the therapy. But nonetheless therapy resulted in a 3-month PFS of only 12.8% with a 6-month OS of 42.7%. Even though the therapy with pazopanib did not show an increased PFS, the authors were able to confirm an early antitumor activity in refractory germ cell tumors. These findings suggest that pazopanib does not seem to be suitable for salvage monotherapy however it might be conceived as an additional factor to other salvage therapeutic options [15].

In addition to pazopanib, two trials suggested the TKI against PDGFR, VEGFR 1–3, c‑KIT, FLT sunitinib to be of clinical benefit in patients with seminomateous or non-seminomateous germ cell tumors refractory to first line therapy. In a phase 2 study, 10 patients with refractory germ cell tumors were enrolled. A total of 5/10 patients received sunitinib 50 mg for four consecutive weeks, followed by a two-week break (4/2). Four of the 5 patients had a tumor marker decline in the 4‑week “on” period, but a tumor marker rise in the 2‑week “off” period, so the other 5 patients received 37.5 mg sunitinib continuously. However, first data showed stable disease (SD) in 4 of the 5 patients in the first group, while all patients developed progressive disease within three cycles of sunitinib. In the group of patients who received 37.5 mg sunitinib continuously, only 1 patient had an overall best response of SD, while the other 4 patients treated on this schedule experienced progressive disease with only one cycle of treatment [16].

Within another phase 2 study, Subbiah et al. treated 5 patients with refractory germ cell tumors after failure of front-line therapy and at least one salvage regimen with sunitinib 50 mg daily in the 4/2 schedule. Of the 5 patients, at least 1 showed PFS for more than 12 weeks. Next generation sequencing of this responding patient showed a RET aberration, which is a possible explanation for the positive treatment response [17].

## Urothelial cancer

Currently chemotherapy and immunotherapy is the mainstay of treatment for metastatic urothelial cancers (mUC) [18]. In addition to standard therapeutic options, antiangiogenic therapy might be considered an alternative or complementary option to conventional cancer treatments.

There is one promising ongoing phase I study assessing the combination of an antiangiogenic agent with a checkpoint inhibitors in refractory mUC. The aim of this study was to examine if combination of cabozantinib, a TKI targeting VEGFR 2 and c‑MET, and nivolumab, an anti-PD-1 inhibitor, work better with or without the monoclonal antibody ipilimumab. A total of 30 patients were treated in 4 dose levels for the combination cabozantinib/nivolumab and 18 patients were treated in 3 dose levels for the combination cabozantinib/nivolumab/ipilimumab. Primary outcome measures of the study were to determine adverse events of the different combinations and the objective response rate (ORR) between the two groups. In both the cabozantinib/nivolumab arm, as well as in the cabozantinib/nivolumab/ipilimumab combination arm no dose limiting toxicities were observed. The most common grade 3–4 adverse events for the cabozantinib/nivolumab and cabozantinib/nivolumab/ipilimumab combination arm were hypertension (23%), neutropenia (17%), lipase enzymes rise (7%), and thrombocytopenia (3%). Regarding the effectiveness, cabozantinib/nivolumab and cabozantinib/nivolumab/ipilimumab combinations showed ORR of 39 (mUC 44%) and 18% (mUC 29%), respectively. So it seems to be of clinical benefit combining cabozantinib and nivolumab with ipilimumab in patients with metastatic genitourinal tumors, especially in mUC [19].

In another phase I study, patients with mUC and FGFR 1-3 mRNA overexpression were treated with the FGFR inhibitor BAY 1163877. First data revealed that in 7/8 treated patients (87.5%) a tumor reduction was observed. Most common adverse events were rising phosphorus levels and diarrhea [20].

Recent data on the combination of neoadjuvant sorafenib (multikinase inhibitor targeting VEGFRs 2, 3, PDGFR-β, c‑Kit, FLT3, and RET) treatment in addition to gemcitabine and cisplatin in 45 patients with mUC demonstrated that additional sorafenib treatment resulted in a pathologic complete response (pT0) in 19 patients (42.2%) altough PFS and median OS were not reached after a median follow-up of 35 months. There were more hematologic grade 3–4 adverse events like platelet count 28.9%, neutrophils 15.6%, hemoglobin levels 4.4% than extrahematologic adverse events [21].

## Conclusion

Currently there are no antiangiogenic agents approved for treatment in genitourinary cancers except mRCC. However, current research suggests that antiangiogenic therapies represent a good and useful addition to conventional oncological treatment options at least in patients where no standard therapeutic options are any more avaiable; therefore, larger multicenter phase III studies are of need to confirm these findings.
